# Sensitive Spectrophotometric Method for Quantitation of Guaifenesin and Dropropizine in Their Dosage Forms

**DOI:** 10.1155/2010/704564

**Published:** 2010-06-29

**Authors:** Ola M. Abdallah

**Affiliations:** Faculty of Pharmacy, October University for Modern Sciences and Arts, El Wahat Road, 6th October City, Egypt

## Abstract

Guaifenesin and dropropizine were analyzed through oxidation with periodic acid to give formaldehyde which was allowed to condense with 4-Amino-5-hydrazino-4H [1,2,4]-triazole-3-thiol (AHTT). The condensation product was further oxidized to yield a purple colored compound with maximum absorption at 550 nm. Beer's law was obeyed in the range of 5–45 *μ*g mL^−1^ for guaifenesin and 10–80 *μ*g mL^−1^ for dropropizine. Both drugs were also successfully determined in their dosage forms.

## 1. Introduction

Guaifenesin (GF), 3-(2-Methoxyphenoxy)-1,2-propanediol; is reported to increase the volume and reduce the viscosity of tenacious sputum and is used as expectorant for productive cough [1]. Different methods have been reported for the analysis of GF including HPLC [2–8], GC [9, 10], capillary electrophoresis mass spectrometry [11], X-ray diffraction [12], voltammetry [13].

Dropropizine (DP), 3-(4-Phenyl-1-piperazinyl)-1,2-propanediol, is a cough suppressant reported to have a peripheral action in nonproductive cough [1]. Only two GC-mass spectrometry methods have been reported for the determination of DP in biological fluids [14, 15] in addition to a manufacturer procedure that involves the determination of dropropizine by measuring its UV absorbance at 237 nm in 0.05 N HCl (personal contact):


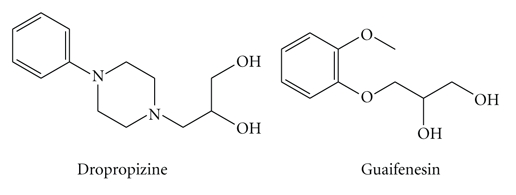


Bromhexine HCl (BR), 2-Amino-3,5-dibromo-N-cyclohexyl-N-methylbenzylamine hydrochloride; N-(2-Amino-3,5-dibromobenzyl)-N-methylcyclohexylamine hydrochloride:


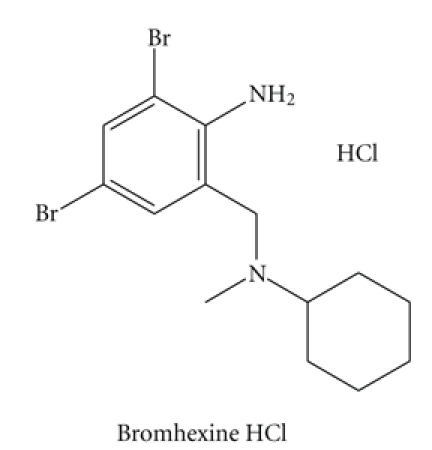


The aim of the present paper is to develop a simple and accurate method for the determination of dropropizine and guaifenesin that permits their analysis in dosage forms without interference from excipients and other coformulated drugs.

## 2. Experimental

### 2.1. Chemicals and Reagents

 Purpald or 4-Amino-5-hydrazino-4H [1,2, 4]-triazole-3-thiol reagent (AHTT) (Sigma-Aldrich) was prepared as 0.5% in 0.5 M hydrochloric acid. Periodic acid (Winlab, UK.) was prepared as 1 mg  mL^−1^ solution in 0.2 M potassium hydroxide. HPLC grade acetonitrile was from Fisher Scientific, UK. Potassium dihydrogen phosphate was from Sigma-Aldrich Chemie, Germany. All other chemicals used were of analytical grade and water was freshly distilled.

### 2.2. Materials

Reference standard guaifenesin (GF), dropropizine (DP) and bromhexine HCl (BR) were kindly supplied by Rameda Co. for pharmaceutical industries and diagnostic reagents, EVA Pharma for pharmaceutical and medical appliances and EVA Pharma for pharmaceutical and medical appliances, respectively.

### 2.3. Pharmaceutical Preparations

**Muclear Capsules:**

It is a product of Rameda Co., Batch No. 08455, claimed to contain 100 mg guaifenesin and 8 mg bromhexine HCl.

**Tussapine Lozenges:**

It is a product of EVA Pharma, Batch No. 602170, claimed to contain 20 mg dropropizine.

### 2.4. Standard Drug Solutions

**Solutions of Guaifenesin:**

0.1 mg  mL^−1^ solution of GF was prepared in distilled water. Aliquots of this solution were diluted to produce working solutions of 5–45 *μ*g mL^−1^.

**Solutions of Dropropizine:**

0.1 mg  mL^−1^ solution of DR was prepared by in distilled water. Aliquots of this solution were diluted to produce working solutions of 10–80 *μ*g mL^−1^.

**Solutions of Bromhexine HCl:**

0.1 mg  mL^−1^ solution of BR was prepared in methanol. Aliquot of this solution was diluted to produce working solution of 50 *μ*g mL^−1^.

### 2.5. Instrumentation

- Shimadzu UV/VIS 1602 Spectrophotometer.
- Agilent 1200 series isocratic quaternary pump HPLC instrument connected to 1200 multiple wavelength UV detector (Germany). Separation was performed on 150 × 4.6 mm Zorbax Extend-C18 column 5 *μ*m particle size (USA). Chromatographic peaks were electronically integrated and recorded using Chemstation software.
- pH/mv Meter with double junction glass electrode (Fisher, USA).

### 2.6. General Procedure

#### 2.6.1. Calibration

 One mL of each working solution of both drugs was transferred in a test tube, then 1 mL periodic acid was added. The mixture was left at room temperature for 15 minutes for GF and 20 minutes for DR, 0.5 mL 5 M KOH solution was then added followed by 1 mL of AHTT solution. The mixture was shaken and allowed to stand for about 15 minutes for GF and 20 minutes for DR. Absorbance of the resulting solution was measured at 550 nm, against blank experiment. Calibration curves relating the absorbance at 550 nm to GF or DR concentrations were plotted and regression analysis of the results was computed.

#### 2.6.2. Assay of Dosage Forms

**Muclear Capsules.**

The well mixed powdered content of five capsules was used in the assay. An amount equivalent to 10 mg of GF was transferred into 100 mL volumetric flask, dissolved in distilled water then adjusted to volume and treated as previously mentioned under calibration procedure.

**Tussapine Lozenges.**

Five tablets were grounded to a homogenous fine powder, weighed and the average mass per tablet was determined. The amount of powder equivalent to 10 mg of DR was dissolved into 70 mL of distilled water. The solution was sonicated for about five minutes then filtered to separate insoluble excipients. Afterwards, the filtrate was accurately collected into 100 mL calibrated flask and diluted to volume with water. The obtained solution labeled to contain 0.1 mg  mL^−1^ of each drug was analyzed by the proposed method as detailed under calibration.

## 3. Results and Discussion

 s-Triazoles have been utilized to produce reagents that can react with drugs containing carbonyl group or susceptible to oxidation with periodic acid to produce carbonyl function such as diols and amino alcohols. In the present work, guaifenesin and dropropizine were converted into formaldehyde and the corresponding carboxylic acids by the selective oxidizing effect of periodic acid. The liberated aldehyde was allowed to react with 4-Amino-5-hydrazino-4H [1,2, 4]-triazole-3-thiol, which is a specific reagent for aldehydic functional group [16]. 

**Scheme 1:**
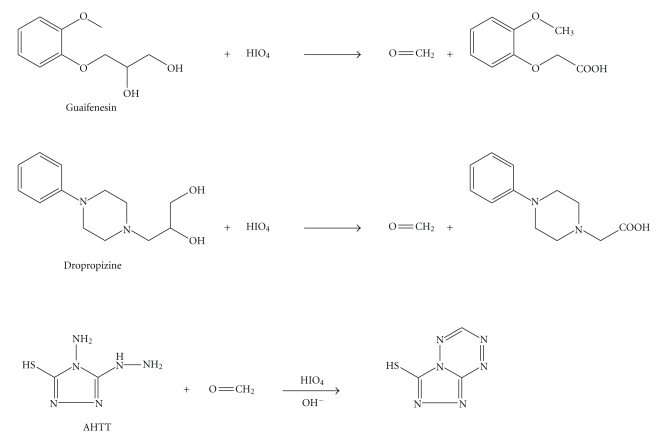
Reactions involved in analysis of guaifenesin and dropropizine.

When AHTT was allowed to condense with formaldehyde followed by treatment with periodic acid and alkali addition, [1,2, 4]-triazolo-[1,2, 4,5] tetrazine-3-thiol colored product was obtained as shown in Scheme 1. 

### 3.1. Optimization of Conditions

 As reported by Jacobsen and Dickinson [16], the reaction involves the addition of alkaline solution of AHTT to the aldehyde solution and aerating the reaction mixture to give a purple-colored product. Mimura et al. [17] modified the procedure of color development by the use of periodic acid as oxidizing agent instead of aeration.

 In the present study, periodic acid has a dual function. It acts as a selective oxidizing agent for polyhydroxy compounds to convert them to formaldehyde and corresponding carboxylic acids and help in the development of the purple colored product according to Mimura et al. [17] modification. It is important to emphasize that Jacobsen and Dickinson [16] used alkaline solution of AHTT (1% in 1 M NaOH) for color production with aldehydes. However, this procedure was modified by using acidic solution of AHTT (0.5% in 0.5 M HCl) which offers two advantages, the first was the use of lower concentration of the reagent; the second was the higher stability of AHTT solution as mentioned by Mimura et al. [17].

**Figure 1:**
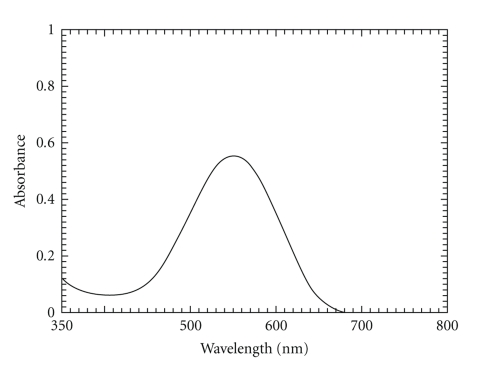
Absorbance spectrum of the colored product produced from the reaction of AHTT and 30 *μ*g mL^−1^ guaifenesin.

**Figure 2:**
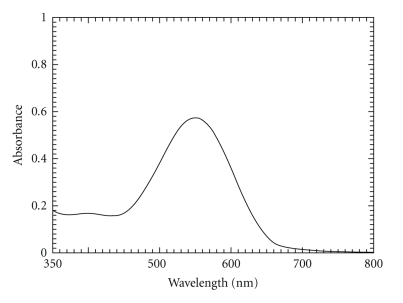
Absorbance spectrum of the colored product produced from the reaction of AHTT and 40 *μ*g mL^−1^ dropropizine.

 As reported for colorimetric determination of some diol-containing drugs [18], solution of guaifenesin and dropropizine was left for some time, then 5 M KOH and AHTT solutions were added whereby a purple color was developed with maximum absorption at 550 nm (Figures 1 and 2). Maximum color intensity was obtained when periodic acid solution was made to react with guaifenesin for 15 minutes and dropropizine for 20 minutes. 

The effect of periodic acid concentration was also studied, it was found to be critical the use of 1 mg  mL^−1^ solution of periodic acid in 0.2 M KOH produces maximum color intensity. Excess acid concentration causes a great decrease in the intensity of the produced color which may be attributed to the strong oxidizing effect of periodic acid on both drugs which may proceed to give further oxidation products.

 The effect of AHTT concentration was also studied where maximum intensity was obtained upon using AHTT solution of 0.5% in 0.5 M HCl. 

 Volume of KOH added was found to be critical; 0.5 mL of 5 M alkali solution was the optimum volume. 

 Shaking of the reaction mixture for 4-5 minutes was essential and produced maximum color intensity after addition of AHTT solution and waiting period of 15 minutes for GF and 20 minutes for DR. The obtained color remained stable for about 40 minutes with both drugs.

### 3.2. Method Validation

#### 3.2.1. Linearity, Detection, and Quantitation Limits

 Calibration curves representing the relation between each drug concentrations and absorbance of colored products were constructed. Results show linear relationship in the range of 5–45 *μ*g mL^−1^ for GF and 10–80 *μ*g mL^−1^ for DR; in triplicate measurement from which linear regression equations were calculated. Correlation coefficient, slope and intercept were listed in Table 1. Results indicate high sensitivity of the proposed method. 

 According to ICH recommendation [19], the approach based on the S.D. of the response and the slope was used for determining the detection and quantitation limits. The theoretical values were assessed practically and given in Table 1.

#### 3.2.2. Accuracy

 Accuracy of the measurements was determined using the calibration standards of two drugs, where mean percentage of 100.58 for GF and 100.22 for DR were obtained, results are shown in Table 1. Accuracy was also assessed by the recovery of added standard, three concentrations each in duplicate to know concentration of dosage forms using the proposed colorimetric method. Results of mean % recovery for added standards in each formulation are reported in Table 2.

#### 3.2.3. Precision

Repeatability and reproducibility of the instrumental response (absorbance of the formed color) were checked during method development and they were assessed from five replicate determinations of sample solutions of GF and DR at the concentration of 30 *μ*g mL^−1^.

#### 3.2.4. Specificity

 The proposed method was applied for the determination of both drugs in their pharmaceutical formulations; results presented in Table 2 revealed that there is no interference from excipients, additives or coformulated drugs such as bromhexine HCl present in Muclear capsules along with guaifenesin. In addition the recoveries of the studied drugs from their formulations were almost the same as the recoveries of the pure added when applying the standard addition technique.

 Results obtained by the proposed method were statistically compared with those obtained from the reported HPLC method for GF [5] and UV manufacturer method for DR. The calculated *t* and *F* values are less than the tabulated ones indicating no significant difference between the proposed and reported methods with respect to accuracy and precision at 95% confidence limit (Table 2).

## 4. Conclusion

 The proposed colorimetric method is selective for polyhydroxy aliphatic compounds, simple and rapid as it takes from 15 to 20 minutes for the sample to be ready for measurement. Validation of the proposed method was carried out according to the ICH guidelines. The short duration of the assay and its specificity were clear bonuses for routine analysis of guaifenesin and dropropizine in their pharmaceutical formulations and in-process quality control.

## Figures and Tables

**Table 1:** Selected spectral data for the determination of guaifenesin and dropropizine by the proposed colorimetric method.

Parameters	GF	DR
Linearity range (*μ*g mL^−1^)	5–45	10–80
Slope ± S.E	0.021 ± 3.7E-04	0.011 ± 1.77E-04
Intercept ± S.E.	0.014 ± 0.0109	0.039 ± 0.0089
Correlation coefficient	0.998	0.998
Accuracy ± S.D. precision	100.58 ± 0.48	100.22 ± 1.36
Injection repeatability (*n* = 15)	0.80–1.26	0.14–0.38
Assay reproducibility intraday (*n* = 9)	Muclear	Tussapine
	0.40%–1.79%	0.13%–0.28%
Interday (*n* = 27)	Muclear	Tussapine
	1.01%–1.72%	0.39%–0.73%
LOQ^a^ (*μ*g mL^−1^)	4	7
LOD^a^ (*μ*g mL^−1^)	2.5	4.5

^a^LOQ and LOD were done practically.

**Table 2:** Statistical analysis of the results obtained by applying the proposed, reported and manufacturer methods for the analysis of GF and DR in their dosage forms.

Preparation	Proposed	Reported	Manufacturer
Muclear	100.34 ± 0.89*	101.18 ± 0.76	—
	*F* = 2.71	—	
	*t* = 0.55	—	
Standard addition	99.39 ± 1.36**		

Tussapine	100.29± 0.76*	—	100.23± 0.84
	*F* =1.55		—
	*t* = 0.33		—
Standard addition	100.60 ± 1.05**		

*Mean of nine determinations (three conc. each in triplet).

**Mean of six determinations (two for each of conc.).
